# VgrG-dependent effectors and chaperones modulate the assembly of the type VI secretion system

**DOI:** 10.1371/journal.ppat.1010116

**Published:** 2021-12-01

**Authors:** Xiaoye Liang, Tong-Tong Pei, Hao Li, Hao-Yu Zheng, Han Luo, Yang Cui, Ming-Xuan Tang, Ya-Jie Zhao, Ping Xu, Tao Dong

**Affiliations:** 1 State Key Laboratory of Microbial Metabolism, Joint International Research Laboratory of Metabolic & Developmental Sciences, School of Life Sciences and Biotechnology, Shanghai Jiao Tong University, Shanghai, China; 2 Department of Immunology and Microbiology, School of Life Sciences, Southern University of Science and Technology, Shenzhen, China; Purdue University, UNITED STATES

## Abstract

The type VI secretion system (T6SS) is a spear-like nanomachine found in gram-negative pathogens for delivery of toxic effectors to neighboring bacterial and host cells. Its assembly requires a tip spike complex consisting of a VgrG-trimer, a PAAR protein, and the interacting effectors. However, how the spike controls T6SS assembly remains elusive. Here we investigated the role of three VgrG-effector pairs in *Aeromonas dhakensis* strain SSU, a clinical isolate with a constitutively active T6SS. By swapping VgrG tail sequences, we demonstrate that the C-terminal ~30 amino-acid tail dictates effector specificity. Double deletion of *vgrG1*&*2* genes (VgrG3^+^) abolished T6SS secretion, which can be rescued by ectopically expressing chimeric VgrG3 with a VgrG1/2-tail but not the wild type VgrG3. In addition, deletion of effector-specific chaperones also severely impaired T6SS secretion, despite the presence of intact VgrG and effector proteins, in both SSU and *Vibrio cholerae* V52. We further show that SSU could deliver a *V*. *cholerae* effector VasX when expressing a plasmid-borne chimeric VgrG with VasX-specific VgrG tail and chaperone sequences. Pull-down analyses show that two SSU effectors, TseP and TseC, could interact with their cognate VgrGs, the baseplate protein TssK, and the key assembly chaperone TssA. Effectors TseL and VasX could interact with TssF, TssK and TssA in *V*. *cholerae*. Collectively, we demonstrate that chimeric VgrG-effector pairs could bypass the requirement of heterologous VgrG complex and propose that effector-stuffing inside the baseplate complex, facilitated by chaperones and the interaction with structural proteins, serves as a crucial structural determinant for T6SS assembly.

## Author summary

Effectors of bacterial secretion systems are generally considered as secreted proteins for interspecies interactions rather than components of the secretion apparatus. Our results reveal the complex interactions of effectors, chaperones, and structural proteins are crucial for T6SS assembly, suggesting an integral role of effectors as parts of the apparatus and distinctive from other secretion systems.

## Introduction

Host-pathogen interaction often involves the translocation of virulence factors by specific protein secretion systems, each considered as a delicate nanomachinery [1]. Their substrate selectivity is a key question for understanding how each system works. Of the six major secretion systems in gram-negative bacteria, the type VI secretion system (T6SS) is of particular interest in host-pathogen interaction for its capability in translocating effectors into a broad range of cell types including bacteria, fungi, and eukaryotic cells [2–5]. The T6SS is widely distributed in gram-negative bacteria including many important human, animal and plant pathogens [6–8]. However, its functions in those diverse species and environments remain largely uncharacterized.

The T6SS comprises a transmembrane anchor, a baseplate and a double tubular sheath-needle structure [7,9–11]. The outer sheath and the inner needle are often made of hundreds of layers of hexametric VipA/B and Hcp, respectively, and are topped by a tip complex consisting of a VgrG trimer and a cone-shape PAAR [9,12,13]. Through sheath contraction, the inner needle is ejected outward, carrying effectors and the tip complex, into the environment or directly into a competing neighbor cell [12,14–16]. Effectors may bind to Hcp, VgrG, and PAAR directly or via a chaperone/adaptor protein [15–21]. In addition, some Hcp, VgrG and PAAR proteins with extended functional domains may directly act as effectors [12,13,22]. Known effectors have exhibited diverse antibacterial and anti-eukaryotic functions including cell-wall hydrolysis, membrane-pore formation, lipases, nucleases, and actin crosslinking toxins [13,14,23–26]. Each antibacterial effector is neutralized by a cognate immunity protein providing specific self-protection [4,14,23,27]. In addition, a number of immunity-independent mechanisms have recently emerged, including formation of kin-only clusters, production of extracellular polysaccharides, envelope stress responses, and general stress responses [28–32]. Although a large number of effectors have been identified, the molecular details of effector selection and loading remain elusive.

VgrG, PAAR and effector proteins are secreted components and yet they also play a vital role in T6SS assembly. For example, of the three VgrG proteins in *V*. *cholerae*, although single deletion of *vgrG1* or *vgrG3* has little effect on T6SS secretion, deletion of *vgrG2* or double deletion of *vgrG1* and *vgrG3* severely impairs secretion [33]. Deletion of all PAAR genes in *Acinetobacter baylyi* abolishes T6SS functions while deletion of PAAR genes in *V*. *cholerae* reduces but not abolishes Hcp secretion and killing efficiency [12]. Combinatorial deletion of effector genes in *V*. *cholerae* inhibits the assembly of contractile T6SS but not the non-contractile mutant, suggesting effectors are important for stabilization but not initiation of T6SS sheath-needle polymerization [14,34]. However, it remains unclear why some VgrG, PAAR and effectors are more important than others in the assembly process.

*Aeromonas dhakensis* is an emerging human pathogen of gastroenteritis and sepsis and its type strain SSU exhibits a constitutively active T6SS [19,35]. Unlike in *V*. *cholerae* and other T6SS organisms that possess multiple extended VgrG and PAAR proteins [13,14,36,37], all VgrG and PAAR proteins in *A*. *dhakensis* SSU contain the conserved structural domain only [38]. The VgrG-specific effectors have been identified, comprising a colicin-like TseC, a nuclease TseI, and a lysozyme-like TseP [19,26,38]. Therefore, the T6SS of SSU provides an ideal model to study the requirement of VgrG and effector proteins in T6SS assembly. In this study, by swapping the C-terminal tail of VgrG proteins, we show that specificity of effector delivery is determined by the C-terminal tail of VgrG proteins. By constructing combinatorial *vgrG* deletion mutants and ectopically expressing chimeric VgrG proteins with different tails to create a one-VgrG/two-effector condition, we show that the previously-known requirement for a heterologous VgrG complex is modulated by the associated effectors. We further show that deletion of chaperone genes could also severely impair T6SS secretion despite the presence of all VgrG and effector proteins. Effector proteins were found to interact with baseplate proteins and TssA in both *A*. *dhakensis* and *V*. *cholerae*. Finally, we propose a VgrG-dependent effector stuffing model illustrating the role of effectors as structural necessity in T6SS assembly.

## Results

### VgrG-mediated effector delivery is highly specific

**Fig 1:**
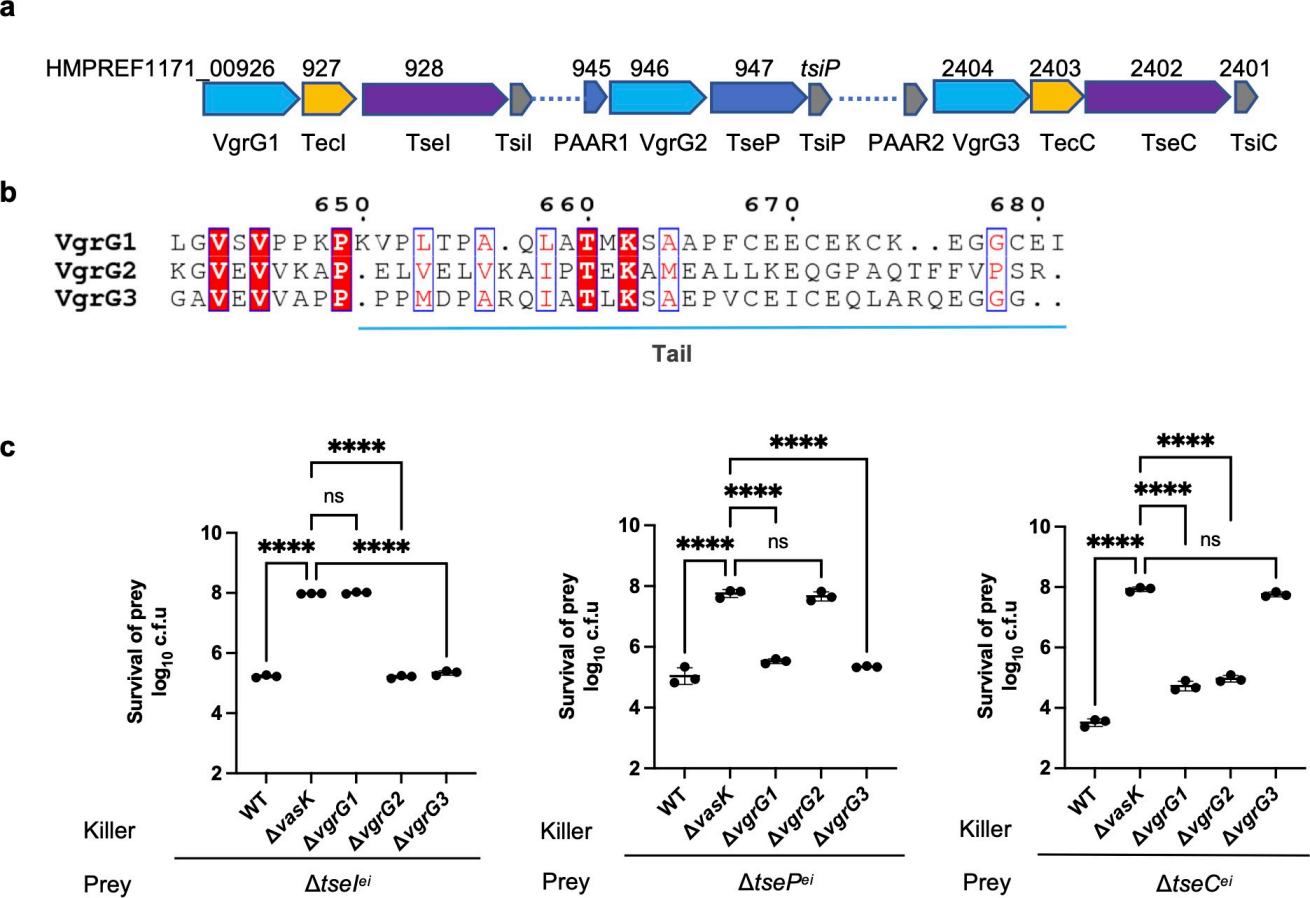
Specificity of VgrG-effector pairs in *A*. *dhakensis* SSU. **A,** Operon structures of the three VgrG-encoding clusters. Gene numbers and proteins are indicated. **B,** Alignment of VgrG sequences highlights the divergence at the C-terminal tail. Alignment was generated using COBALT and visualized using the ESPript server. Full sequence alignment is shown in Figure A in S1 Text. **C,** Competition assay of wild type (WT), the T6SS null Δ*vasK* mutant, and individual Δ*vgrG* mutants against effector-immunity deletion mutants. Survival of prey was quantified after co-incubation with the killer strains. Error bars indicate the mean +/- standard deviation of at least three biological replicates and statistical significance was calculated using one-way ANOVA analysis. ****P < 0.0001, ns: not significant.

Of the three known effectors in *A*. *dhakensis* SSU, each resides in an operon containing an upstream *vgrG* (Fig 1A). All three VgrG proteins are highly conserved with an N-terminal hallmark VgrG signature but the C-terminal ends (~ 30 amino acids) of the VgrGs are divergent (Fig 1B and A in S1 Text). Using bacterial competition assays of single *vgrG* deletion mutants against effector-immunity mutants, we found that deleting any of the three *vgrG* genes abolished the killing of its cognate downstream effector-immunity mutant but not the other two immunity mutants (Fig 1C). These results confirm the known VgrG-dependence of each effector and further show that such dependence is highly specific.

### VgrG C-terminal divergent tail dictates effector delivery specificity

**Fig 2:**
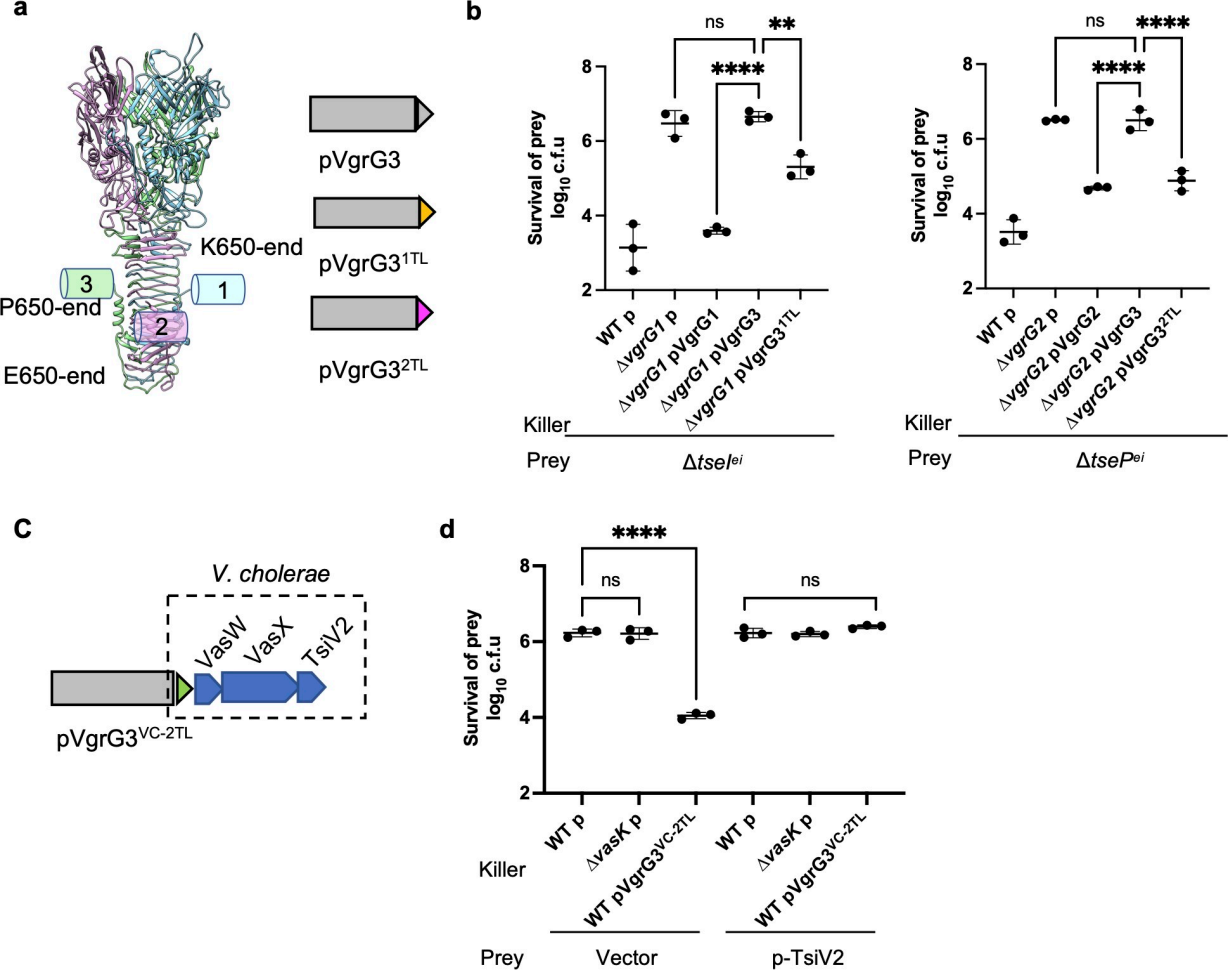
VgrG C-terminal tails dictate effector specificity. **A,** Schematic of tail swapping of VgrG proteins. Structural model of the VgrG complex was generated using the VgrG1 structure of *P*. *aeruginosa* as template (PDB: 6H3L) in Chimera. Chimeric VgrG3 proteins with the tail sequence of VgrG1(K650 to end) or VgrG2(E650 to end) were constructed and expressed on pBAD vectors. **B,** Competition assay of *vgrG* mutants expressing chimeric VgrG proteins against the cognate immunity-defective mutants. WT and *vgrG* mutants were transformed with an empty pBAD plasmid (p) or VgrG-encoded plasmids as indicated. Killer and prey strains were co-incubated on LB plates with 0.01% arabinose and survival of prey was enumerated by serial dilutions and plating on selective media. **C**, Chimeric VgrG3 with the VasX effector module of *V*. *cholerae*. A pBAD plasmid expressing chimeric VgrG3 with the C-terminal tail of *V*. *cholerae* VgrG2 and its downstream chaperone (VasW), effector (VasX) and immunity (TsiV2) was constructed. **D,** Competition assay of WT expressing an empty plasmid (p) or the chimeric VgrG against an SSU mutant prey expressing an empty vector or the immunity protein TsiV2. The Δ*vasK* mutant serves as a T6SS null control, and the prey strain is the Δ*vgrG1&3* double mutant. For B&D, error bars indicate the mean +/- standard deviation of at least three biological replicates and statistical significance was calculated using one-way ANOVA analysis. **P < 0.01, ****P < 0.0001, ns: not significant.

It has been previously shown that the C-terminal tail of VgrG proteins determines the specificity of VgrG-effector interaction in a number of species including *Agrobacterium tumefaciens*, enteroaggregative *Escherichia coli*, *V*. *parahaemolyticus*, and *Pseudomonas aeruginosa* [20,39–42]. To test how effectors are specifically recognized by cognate VgrGs in SSU, we swapped the VgrG3 tail sequence (P650 to end) with the ones from VgrG1 (K650 to end) and VgrG2 (E650 to end) to test whether these chimeric VgrG3 variants can functionally complement the deletions of the corresponding *vgrGs* (Fig 2A). Results show ectopic expression of VgrG3^1TL^ (VgrG3 N-terminal with VgrG1 tail) partially restored the killing activity of Δ*vgrG1* mutant against its corresponding immunity defective mutant Δ*tseI*^*ei*^ (Fig 2B). Similarly, ectopic expression of VgrG3^2TL^ (VgrG3 N-terminus with VgrG2 tail) functionally complemented the Δ*vgrG2* mutant (Fig 2B) by restoring its killing activity against the corresponding immunity defective mutant Δ*tseP*^*ei*^.

Next, we tested whether VgrG-tail swapping could deliver an effector of another species. This is different from our recent report that an effector from *A*. *dhakensis* was delivered by the T6SS of *V*. *cholerae* as a hybrid fusion to the PAAR2 protein [43]. We expressed VasX, a T6SS effector in *V*. *cholerae*, and its associated chaperone VasW and immunity protein TsiV2 in SSU (Fig 2C). Successful delivery of VasX would allow SSU to kill its sister cells that do not express the VasX-specific immunity protein TsiV2. Intraspecies competition analysis shows that SSU expressing the VgrG3^VC-2TL^-VasW-VasX-TsiV2 plasmid could outcompete the Δ*vgrG1&3* mutant carrying the pBAD empty vector but not the pBAD-TsiV2 vector (Fig 2D). By contrast, wild type SSU failed to outcompete the Δ*vgrG1&3* mutant since it encodes a full set of immunity proteins to native T6SS effectors. These results collectively indicate that swapping the C-terminal tail sequence of VgrG proteins enables delivery of VgrG-dependent effectors of the same or different species.

### Double deletion of *vgrG* genes abolishes T6SS secretion

**Fig 3:**
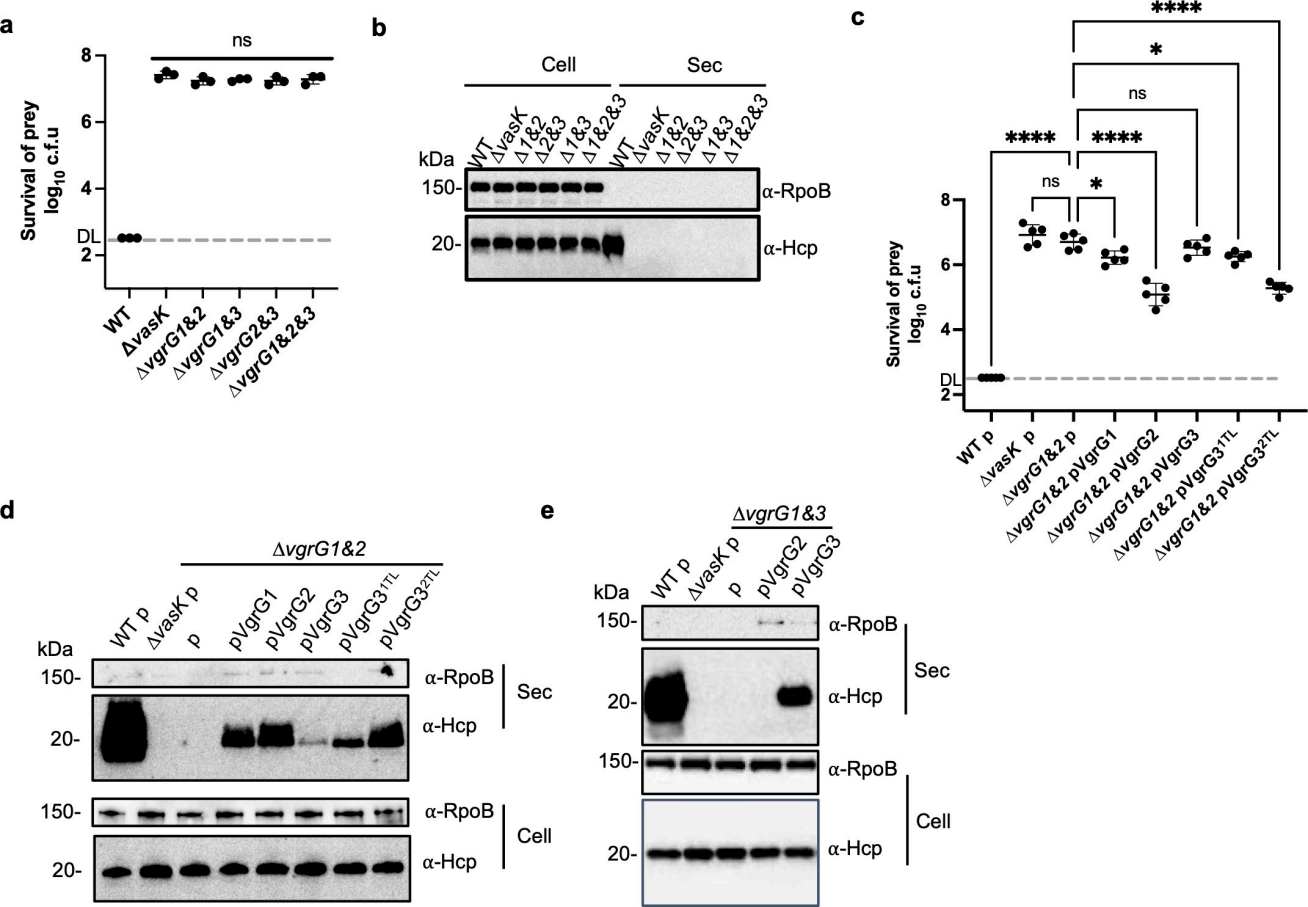
Chimeric VgrG proteins restore T6SS secretion in Δ*vgrG* mutants. **A,** Competition analysis of double and triple *vgrG* deletion mutants against *E*. *coli*. Survival of *E*. *coli* was quantified by serial dilutions on selective medium. When no surviving *E*. *coli* was detected in the dilution series, one colony was artificially counted at the lowest dilution factor for calculation and statistical analysis, representing the detection limit (DL, grey dashed line). DL may vary depending on the plating volume of each assay. **B,** Western blot of Hcp secretion in the *vgrG* deletion mutants. Secreted proteins were resolved by SDS-PAGE and detected by Western blot analysis with anti-Hcp and anti-RpoB antisera. Hcp is a hallmark of T6SS secretion while RpoB, the RNA polymerase beta-subunit, serves as a control for equal loading (Cell) and cell lysis in secretion samples (Sec). **C,** Functional complementation of chimeric VgrG proteins in competition analysis. Killer strains are SSU WT and mutant strains transformed with the empty pBAD vector or vectors expressing different VgrG variants as indicated. Killer strains and the *E*. *coli* prey were co-incubated on LB media with 0.01% arabinose and survival of *E*. *coli* was quantified by serial dilutions and plating on selective media. DL: detection limit. **D,** Western blot of Hcp secretion. Strains were transformed with an empty pBAD vector (p) or vectors expressing VgrG proteins as indicated. Expression of VgrG proteins was induced by the addition of arabinose to aerobically grown LB culture. **E.** Expression of plasmid-borne VgrG3 restores Hcp secretion in the Δ*vgrG1&3* mutant background. Strains expressing an empty pBAD vector (p) or VgrG proteins are indicated. Western blot of Hcp and RpoB in the whole cell (Cell) and secreted (Sec) samples was performed similarly as in **B** and **D**. Hcp and RpoB were detected in whole cell (Cell) and secreted (Sec) samples. For **A**&**C**, error bars indicate the mean +/- standard deviation of at least three biological replicates and statistical significance was calculated using one-way ANOVA analysis. *P < 0.05, ****P < 0.0001, ns: not significant.

Previous findings indicate that a heterotrimeric VgrG complex is required for T6SS functions in *V*. *cholerae* [33], in which two of the three VgrG proteins, VgrG1 and VgrG3, are “evolved” VgrG effectors with C-terminal extended functional domains [13]. Importantly, VgrG2, the one without an extended domain, seems to be more important than the other two VgrG proteins since its deletion abolishes T6SS secretion [33]. By contrast, the three SSU VgrG proteins do not possess any extended domains (Fig A in S1 Text). We then tested whether a heterotrimeric VgrG complex is required in SSU. By constructing a series of combinatorial *vgrG* deletion mutants, we found that none of the double or triple *vgrG* deletion mutants was able to kill a competing *E*. *coli* prey or to secrete Hcp (Fig 3A and 3B), suggesting that a VgrG homotrimer comprising only one VgrG protein is insufficient for T6SS secretion.

### The VgrG-heterotrimer requirement is dependent on effectors

Having built a set of chimeric VgrG and effector pairs and a set of *vgrG* deletion mutants, we next used them to investigate why a single VgrG cannot support T6SS secretion. Using the functional VgrG3^1TL^ and VgrG3^2TL^ chimeric plasmid-borne constructs that can deliver TseI and TseP, respectively, we expressed them in the Δ*vgrG1&2* (VgrG3^+^ only) mutant so that it would have VgrG3 carrying its native TseC with an additional effector TseI or TseP. As control, we also expressed plasmid-borne wild-type VgrG proteins in the T6SS-inactive Δ*vgrG1&2* mutant. Using a competition assay of *vgrG* mutants against an *E*. *coli* prey, we found that expression of VgrG1 and VgrG2 but not VgrG3 or vector only partially restored the killing activities of the Δ*vgrG1&2* mutant, suggesting again a heterotrimeric VgrG complex is required (Fig 3C). Importantly, one of the VgrG3 hybrids, VgrG3^2TL^, complemented the Δ*vgrG1&2* mutant in the bacterial competition assay as efficiently as the pVgrG2 plasmid. A moderate complementation effect was also observed for VgrG3^1TL^.

Because bacterial competition could be affected by effector functions, we next tested Hcp secretion in these strains to compare T6SS activities using Western blotting analysis (Fig 3D). As expected, there was no secreted Hcp detected in the Δ*vgrG1&2* or the Δ*vasK* mutant. Ectopic expression of VgrG1, VgrG2, and VgrG3^2TL^ in the Δ*vgrG1&2* mutant substantially increased Hcp secretion. By contrast, expression of VgrG3 resulted in a background level of Hcp secretion, which was detectable only after much longer exposure. Expression of the VgrG3^1TL^ resulted in an intermediate level of Hcp secretion. These results are in general agreement with the competition analysis.

To determine if this phenotype is specific to the Δ*vgrG1&2* and to test if expression of VgrG3 is functional, we also ectopically expressed VgrG2 and VgrG3 in the Δ*vgrG1&3* mutant. Again, using Western blotting analysis, we found that induced expression of VgrG2 failed to restore Hcp secretion in the Δ*vgrG1&3* (VgrG2^+^) mutant while that of VgrG3 did (Fig 3E). Collectively, the results of expressing VgrG3-tail variants in combinatorial *vgrG* deletion mutants suggest that T6SS assembly requires the presence of multiple effectors but not a heterotrimeric VgrG complex.

### Effector-cognate chaperones are crucial to T6SS secretion

**Fig 4:**
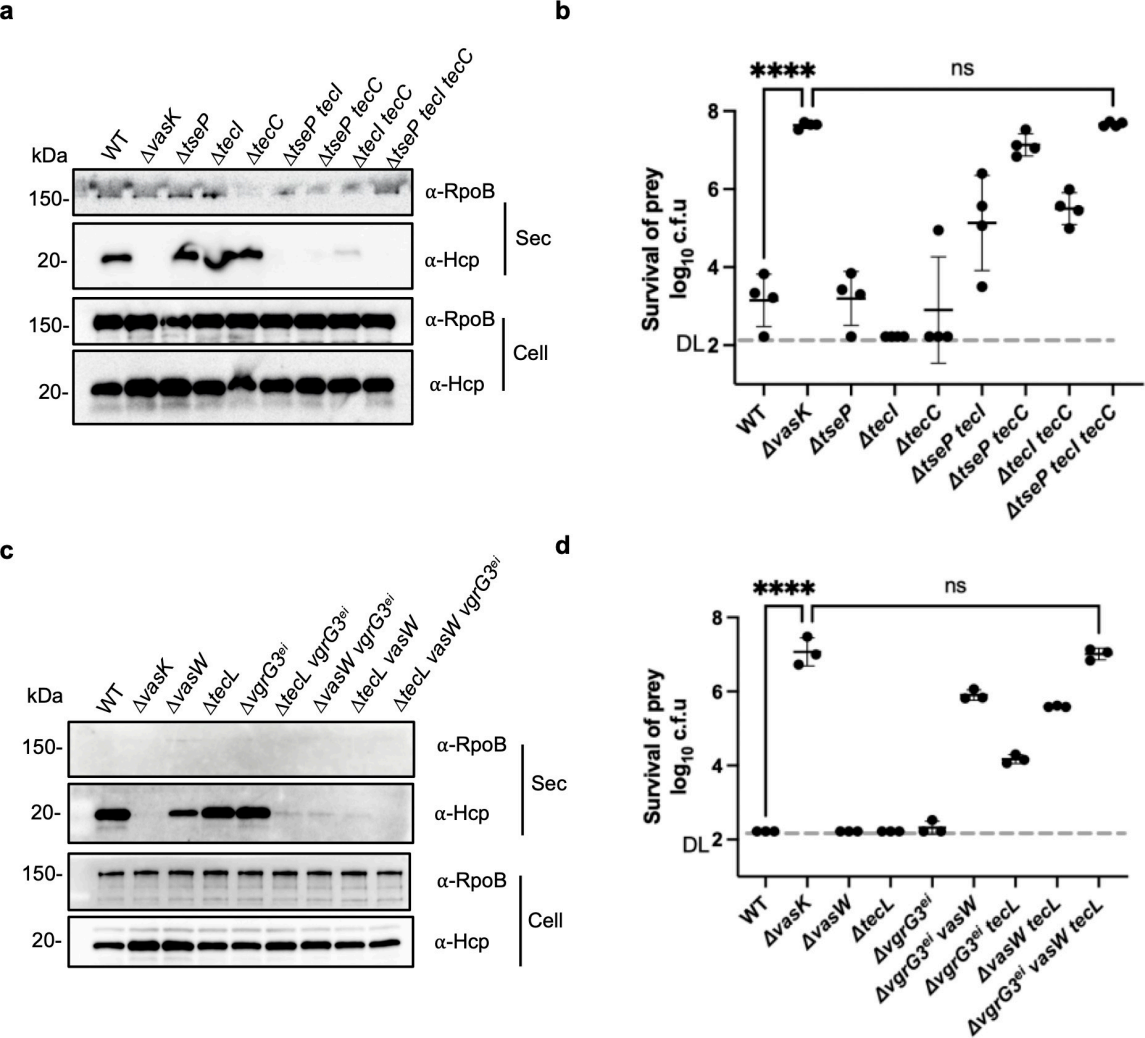
Effects of chaperone gene deletions on T6SS secretion. **A,** Western blot of Hcp secretion in chaperone deletion mutants. Chaperone genes *tecI* and *tecC* were deleted individually or in combination with the chaperone-independent effector gene *tseP*. Secreted samples were collected from aerobically growing cultures. Whole cell (Cell) and secreted samples (Sec) were subject to SDS-PAGE and Western blotting analyses. Signals were detected using anti-RpoB and anti-Hcp antibodies, respectively. The Δ*vasK* mutant serves as a T6SS null control. **B,** Competition analysis of chaperone gene deletion mutants against the *E*. *coli* prey. Error bars indicate the mean +/- standard deviation of four biological replicates. **C,** Western blot of Hcp secretion in chaperone deletion mutants of *V*. *cholerae*. Chaperone genes *vasW* and *tecL* were deleted individually or in combination with the *vgrG3* effector-immunity gene pair Δ*vgrG3*^*ei*^. VgrG3 is a chaperone-independent effector in *V*. *cholerae*. Signals were detected using anti-RpoB and anti-Hcp antibodies, respectively. **D,** Competition analysis of *V*. *cholerae* chaperone gene deletion mutants against the *E*. *coli* prey. Error bars indicate the mean +/- standard deviation of three biological replicates. For **B** and **D**, the killer-to-prey ratio is 10:1, and statistical significance was calculated using one-way ANOVA analysis. ****P < 0.0001, ns: not significant. DL: detection limit.

Because our previous results show that chaperones are required for secretion of effectors TseI and TseC [19,26], we next tested whether chaperone genes are required for T6SS secretion. SSU genome encodes two TEC(DUF4123)-domain chaperones TecI and TecC, respectively. We constructed combinatorial deletions of *tecI* and *tecC* with the chaperone-independent effector gene *tseP* and tested their effects on Hcp secretion and bacterial competition against the *E*. *coli* prey. Western blotting analysis shows that, while single gene deletion had little effect on Hcp secretion, double deletion of both chaperone genes substantially reduced Hcp secretion (Fig 4A). Interestingly, when deletion of *tseP* was introduced to chaperone gene deletion mutants, Hcp secretion was severely impaired while cytosolic Hcp levels were not affected. Competition assays against an *E*. *coli* prey show consistent results that combinatorial deletions of chaperone genes with or without *tseP* all resulted in significantly reduced killing ability against *E*. *coli* (Fig 4B).

Next, we tested whether the chaperone gene requirement also occurs in *V*. *cholerae* by constructing deletion mutants of chaperone genes in *V*. *cholerae* strain V52 [2,14]. We have previously found that the triple deletion of *tseL*, *vasX* and *vgrG3* genes abolished T6SS secretion [34]. There are two TEC chaperones, TecL and VasW, that are required for the secretion of effectors TseL and VasX, respectively [19,21]. Using bacterial competition and Hcp secretion assays, we found that deletion of chaperones severely impaired T6SS functions (Fig 4C and 4D). When either chaperone deletion was introduced to the *vgrG3*^*ei*^ mutant, a *vgrG3*-null background lacking both *vgrG3* and its immunity gene *tsiV3*, T6SS secretion was also impaired. Collectively, these results show that chaperones that facilitate VgrG-effector interaction are also crucial for T6SS assembly.

Therefore, the observed VgrG-heterotrimer requirement for T6SS assembly is modulated by efficient loading of heterogeneous effectors to different VgrG proteins, and the presence of effectors and VgrG proteins without chaperones is severely inefficient to support T6SS assembly.

### Effectors directly interact with structural proteins

**Fig 5:**
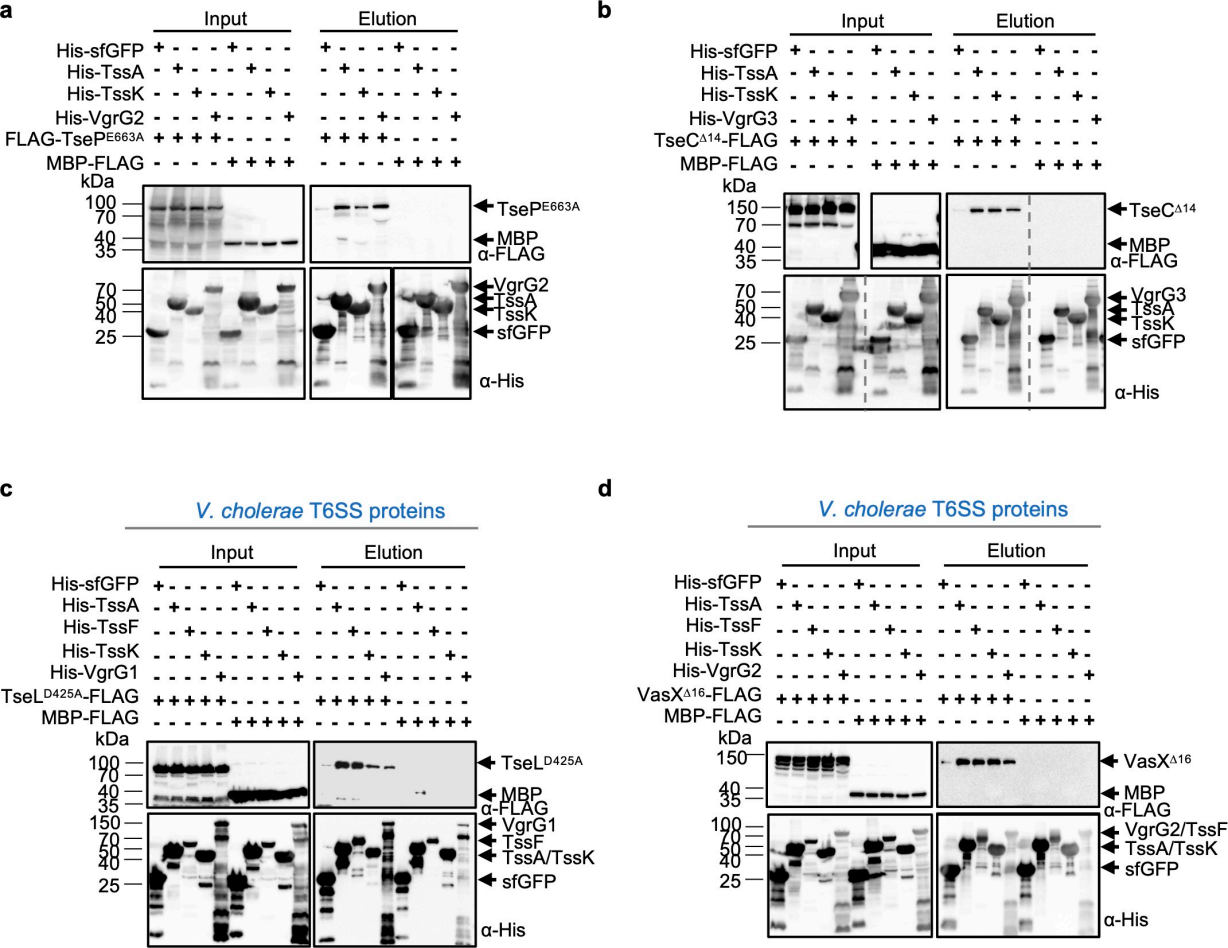
Interaction of effectors with baseplate proteins. **A**, Pull-down analysis of structural proteins and TssA with the catalytically inactive effector TseP^E663A^. **B**, Pull-down analysis of structural proteins and TssA with TseC^Δ14^. **C**, Pull-down analysis of *V*. *cholerae* structural proteins and TssA with the catalytically inactive effector TseL^D425A^. **D**, Pull-down analysis of *V*. *cholerae* structural proteins and TssA with the colicin-inactive effector VasX^Δ16^. For all pull-down assays, effectors carry a C-terminal or an N-terminal FLAG tag as indicated and bait proteins are fused with an N-terminal 6His tag. His-sfGFP and MBP-FLAG serve as a negative control for nonspecific interaction. Expression of all proteins was induced in *E*. *coli* individually, and cell lysates were mixed in pairs. Samples were detected by Western blotting analysis using the anti-FLAG and anti-His antibodies, respectively. All pull-down experiments were performed at least twice and a representative result is shown.

To determine how effectors contribute to T6SS assembly, we used pull-down analysis to test the interaction of effectors with the baseplate proteins TssE/F/G/K, as well as with the assembly chaperone TssA that interacts with multiple structural proteins [44–48]. All proteins were individually expressed in *E*. *coli* and cell lysates were mixed in pairs. A preliminary pull-down analysis between different baseplate proteins and effectors (or their catalytically inactive mutants where indicated) suggested that effectors may interact with TssK and TssA (Fig B in S1 Text). Interaction with TssE, TssF, or TssG was inconclusive due to poor expression or affinity enrichment. Therefore, we focused on TssK and TssA in the follow-up assays. Pull-down analyses showed positive interaction between His-tagged TssK and TssA with the corresponding FLAG-tagged effectors, TseP and TseC. His-tagged superfolder green fluorescent protein (sfGFP) and FLAG-tagged maltose binding protein (MBP) serve as the negative control while the effector-specific VgrG proteins serve as the positive control, which collectively show the observed effector interactions were specific (Fig 5A and 5B).

To test if the interaction between effectors and baseplate proteins also occurs in *V*. *cholerae*, in which effectors are also critical to the T6SS assembly [34], we performed similar pull-down analyses using *V*. *cholerae* T6SS proteins. A preliminary analysis testing TssE/F/G/K and TssA with effectors suggested positive interactions between effectors with TssF/K and TssA (Fig C in S1 Text). Results with TssE and TssG were again inconclusive due to reduced expression or affinity enrichment. By focusing on TssA/F/K as bait proteins, we show that these His-tagged baits could pull down FLAG-tagged *V*. *cholerae* effectors, TseL and VasX (Fig 5C and 5D). Control samples using His-sfGFP, FLAG-MBP, and effector-specific His-VgrG proteins exhibited negative and positive interactions as expected.

Collectively, these results reveal that effectors likely participate in the assembly process by interacting not only with VgrG for secretion but also with other non-secreted structural proteins and the assembly chaperone TssA.

## Discussion

As a widespread molecular weapon among gram-negative pathogens, the T6SS is analogous to a speargun with its spear loaded onto the trans-membrane-baseplate complex as the main frame and ejected by a contractile sheath as the spring cord. In the middle of the baseplate and on top of the sheath-tube structure sits the VgrG spike complex as the spearhead. This central position highlights its involvement in formation of the pre-firing complex and initiation of sheath-tube polymerization. However, the molecular details that govern T6SS assembly still remain elusive. Specifically, why certain VgrG proteins are more critical than others despite of near identical conserved sequences [13,33,49] and why VgrG-dependent effectors are required for T6SS assembly [34,50]?

**Fig 6:**
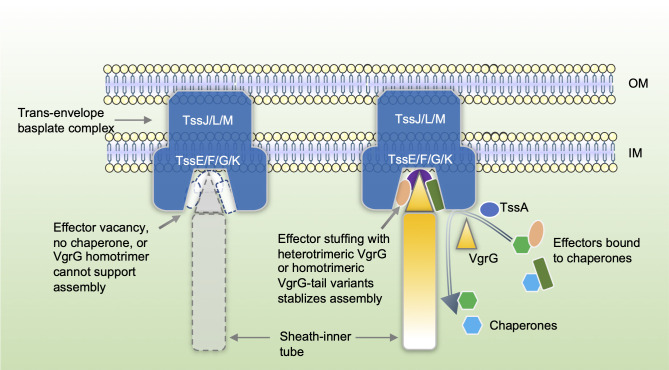
Effectors serve as structural components for T6SS assembly. Effector vacancy, chaperone deletion, or VgrG homotrimer cannot support T6SS assembly. The assembly of T6SS in *A*. *dhakensis* requires multiple effectors bound to a heterotrimeric VgrG spike or a homotrimer of VgrG hybrids with varied C-terminal tails, as well as sufficient effector-baseplate interaction. The process involves effector-chaperone interaction for stabilizing and delivering effectors to the VgrG spike, as well as direct interaction between effector and structural proteins. Therefore, the VgrG-dependent effectors might be considered integral parts of the assembly.

Here we use a simple VgrG model in *A*. *dhakensis* SSU in which all three VgrG proteins contain canonical VgrG-domains only and are near identical except for the C-terminal tail sequences [38]. This is advantageous in comparison with previous research in *V*. *cholerae* and other species, which may be complicated by the presence of multiple evolved VgrG proteins that function not only as a structural component but also as effectors through their C-terminal extended domains [13,51]. We show that swapping the C-terminal tail could alter VgrG-effector specificity not only among SSU effectors but also could enable SSU to deliver a *V*. *cholerae* effector VasX. Furthermore, combinatorial deletion of *vgrG* genes abolished T6SS function, which allows us to use chimeric VgrG proteins to construct a one-VgrG only condition but delivering two different effectors. Results show that the VgrG3-only mutant ectopically expressing a VgrG3-tail chimera but not the wild type VgrG3 is T6SS-active. In addition, deletion mutants lacking chaperones that facilitate effector-VgrG binding also are impaired in T6SS secretion. This observation is consistent with a previous report that double deletion of chaperone genes abolished the T6SS secretion in *A*. *tumefaciens* [50]. These results collectively indicate that effectors, but not the VgrG proteins per se, are the key factors dictating the requirement for heterologous VgrG-spike complex previously observed in *V*. *cholerae* [13,33] and here in SSU (Fig 6).

While double *vgrG* deletion mutants abolished T6SS secretion in SSU (Fig 3A and 3B), single deletion of *vgrG* did not, indicating none of the VgrG proteins is required for T6SS assembly. Similarly, our previous results find none of the effectors is required for T6SS [19,26,38]. In addition, expression of *vgrG3* in the VgrG3^+^-only mutant cannot restore T6SS activities (Fig 3C and 3D), and the VgrG2^+^-only mutant cannot be complemented by induced expression of VgrG2 (Fig 3E). These results indicate that the loss of T6SS activities in the single VgrG^+^-only mutant is not due to an insufficient expression level of VgrG proteins.

Here we propose a model depicting that the effector-stuffing effect inside the baseplate cavity is required for T6SS assembly (Fig 6). This is also built on the previous observations that effectors are involved in T6SS assembly since combinatorial deletion of all effector genes in *V*. *cholerae* and *A*. *dhakensis* SSU abolishes T6SS secretion [34,38]. In addition, a recent report has also shown that the T6SS secretion in *Enterobacter cloacae* requires the presence of two VgrG-dependent Rhs-family effectors RhsA and RhsB, and the N-terminal PAAR domain of RhsA was sufficient for stabilizing its interacting VgrG trimer but not for restoring T6SS secretion in the Δ*rhsA* Δ*rhsB* mutant, highlighting the required physical presence of the full length RhsA [52]. In the absence of effectors, only non-contractile T6SS sheath-tube can be formed in *V*. *cholerae* [34], suggesting that effectors are not required for initiating sheath-tube assembly but critical for stabilizing polymerization and preventing from premature contraction. Likely due to VgrG-effector specific interaction, lacking effectors, chaperones, and specific VgrG proteins all lead to similar effects to effector-deletions (Fig 6). In addition, the pull-down analysis suggests that effectors can also interact with T6SS baseplate and the TssA chaperone, suggesting such interactions may be important for recruiting the effectors to the baseplate and for stabilizing the structures. We propose that the spike-associating VgrG-dependent effectors might be considered as integral structural components of T6SS beyond their known functions in interspecies interactions. Identification of the effector-baseplate interaction will lead to future work involving high-resolution analyses, including Cryo-electron microscopy and tomography analyses, to elucidate the molecular details of these interactions both *in vitro* and in cells and the resulting effects on the T6SS assembly and effector secretion.

Lastly, delivery of heterologous cargo proteins will greatly expand the application of T6SS in a number of biotechnological and therapeutic areas. However, it is hindered by our limited understanding of effector delivery, and one main challenge is due to the complex and specific binding of effectors to their cognate T6SS carrier proteins. VgrG-mediated heterologous effector delivery has only been demonstrated in the form of fusion proteins [34,53–55]. Here we were able to use a chimeric VgrG-tail construct to deliver a heterologous effector of *V*. *cholerae* by the SSU T6SS, demonstrating as proof-of-concept for the delivery of a heterologous and standalone cargo protein that can be further explored for biotechnological applications in future studies.

## Methods

### Bacterial strains and growth conditions

Strains, plasmids, and primers used in this study are listed in Table A in S1 Text and available upon request. Cultures were routinely grown in Lysogeny Broth ([w/v] 1% tryptone, 0.5% yeast extract, 0.5% NaCl) aerobically at 37°C or 30°C as indicated. The following antibiotics were used: streptomycin (100 μg/ml), ampicillin (100 μg/ml), kanamycin (50 μg/ml), chloramphenicol (25 μg/ml for *E*. *coli*, 2.5 μg/ml for *A*. *dhakensis* SSU and *V*. *cholerae* V52).

### Competition assay

Cultures of killer and prey strains were grown in liquid LB to exponential phase (OD_600_ = 1) and stationary phase (OD_600_ = 2), respectively. Cells were collected by centrifugation and resuspended in LB. Killer and prey cells were mixed at a ratio of 5: 1, spotted on LB-agar plates, and incubated for 3 h at 37°C. The mixture was retrieved in 500 μL LB in 2 ml tubes. After vigorous shaking, a series of 10-fold dilutions was plated on selective plates with antibiotics. The mean Log_10_ c.f.u of recovered cells was plotted and error bars show mean +/- standard deviation of at least three biological replicates. One-way ANOVA analysis was performed using the Prism software with default settings.

### Protein secretion assay

Aerobically grown cultures were grown in LB at 30°C to OD_600_ = 1. Cells were centrifuged at 2,500 × g for 8 min and then resuspended in fresh LB. Gene expression on pBAD vectors was induced with 0.01% [w/v] L-arabinose at 30°C for 1 h. Cultures were centrifuged at 10,000 × g at room temperature, and the resulting supernatant samples were centrifuged again at 10,000 × g to remove any residue cells. TCA (trichloroacetic acid) was added to the supernatants at a final concentration of 20% [v/v] for protein precipitation. Proteins were collected at 15,000 × g for 30 min at 4°C and pellets were washed with acetone at room temperature and air-dried. Both whole cell and proteins samples were resuspended in SDS-loading dye and boiled for 10 min before SDS-PAGE analysis.

### Western blotting analysis

Whole cell and secreted proteins were subject to SDS-PAGE analysis, after which resolved proteins were transferred by electrophoresis to a PVDF membrane (Bio-Rad). A solution with 5% [w/v] non-fat milk in Tris-buffered saline with Tween-20 (TBST) buffer (50 mM Tris, 150 mM NaCl, 0.1% [v/v] Tween-20, pH 7.6) was used to block the PVDF membrane for 1 h at room temperature. Primary and secondary HRP-conjugated antibodies were sequentially used to treat the PVDF membrane, after which the Clarity ECL solution (Bio-Rad) was used for signal detection. Antibodies were purchased from Biolegend (RpoB, Product # 663905), Thermo Scientific (V5, Product # 37–7500), ABclonal (FLAG, Product # AE005 and 6His, Product # AE003), ZSGB-Bio (Product # ZB-2305 (mouse) and # ZB-2301 (rabbit)). The polyclonal custom antibody to Hcp was made by Shanghai Youlong Biotech.

### Pull-down analysis

Genes of interest were cloned into pET and pBAD vectors with His, 3V5 or FLAG epitope tags and expression was induced in *E*. *coli* individually. Cells were grown in LB with appropriate antibiotics to OD_600_ of 0.6–0.8, and induced with 1 mM IPTG for 18 h at 20°C for pET vectors and with 0.1% arabinose for 3 h at 30°C for pBAD vectors. Pellets were collected by centrifugation, resuspended in lysis buffer (20 mM Tris, 500 mM NaCl, 50 mM imidazole, pH 8.0 with protease inhibitor (Thermo Scientific)), and lysed by sonication. After centrifugation to remove cell debris, supernatants were mixed as input samples. Samples were loaded to Ni-NTA resin (Smart-lifesciences), then washed 4–5 times with wash buffer (20 mM Tris pH 8.0, 500 mM NaCl, 50 mM imidazole), and eluted in elution buffer (20 mM Tris pH 8.0, 500 mM NaCl, 500 mM imidazole). Input and elution samples were analyzed by Western blot. Expression of V5-tagged TseP and FLAG-tagged TseI was unstable and excluded in the pull-down analysis.

### Bioinformatics analysis

Gene sequences of SSU were retrieved from the draft genome assembly (GenBank NZ_JH815591.1). VgrG protein sequences were aligned using COBALT [56] and Clustal Omega [57] and visualized using ESPript with default settings (https://espript.ibcp.fr)[58]. VgrG structural models were generated using the Phyre2 program [59]. Chimera [60] was used to visualize and compare VgrG predicted models with the VgrG1 of *Pseudomonas aeruginosa* structure model (PDB: 6H3L) [61].

## Supporting information

S1 Text Supplemental figures and table for additional data and strain information. **Fig A. Sequence alignment of VgrG proteins in *A*. *dhakensis* SSU.** The three VgrG proteins were aligned using the BLAST Multiple Alignment tool. Alignment was downloaded in Clustal format and visualized using ESPript with default settings (https://espript.ibcp.fr). The predicted structure of VgrG1, generated by Phyre2, was used as structural template in ESPript. **Fig B. Pull-down analyses of *A*. *dhakensis* effector-structural protein interactions. a**, Pull-down analysis of structural proteins with the catalytically inactive TseI^HFH-AAA^. The full-length protein and the cleaved C-terminus of TseI are indicated. **b**, Pull-down analysis of structural proteins with the effector TseC. **c**, Pull-down analysis of TssA with TseI^HFH-AAA^. **d**, Pull-down analysis of TssA with TseC. For all pull-down assays, effectors carry a C-terminal 3V5 tag and bait proteins are fused with an N-terminal 6His tag. His-sfGFP serves as a negative control for nonspecific interaction. All proteins were individually expressed in *E*. *coli* and cell lysates mixed in pairs. Samples were detected by Western blotting analysis using the anti-V5 and anti-His antibodies, respectively. All pull-down experiments were performed at least twice and a representative result is shown. **Fig C. Pull-down analyses of *V*. *cholerae* effector-structural protein interactions. a,** Pull-down analysis of *V*. *cholerae* structural proteins with the catalytically inactive TseL^D425A^. **b,** Pull-down analysis of *V*. *cholerae* structural proteins with the effector VasX. **c**, Pull-down analysis of *V*. *cholerae* TssA with TseL^D425A^. **d**, Pull-down analysis of *V*. *cholerae* TssA with VasX. For all pull-down assays, effectors carry a C-terminal 3V5 tag and bait proteins are fused with an N-terminal 6His tag. His-sfGFP serves as a negative control for nonspecific interaction. All proteins were individually expressed in *E*. *coli* and cell lysates mixed in pairs. Samples were detected by Western blotting analysis using the anti-V5 and anti-His antibodies, respectively. All pull-down experiments were performed at least twice and a representative result is shown. **Table A. Plasmids, strains and primers.**
(DOCX)
