# Supplementary material for: VgrG-dependent effectors and chaperones modulate the assembly of the type VI secretion system
Source: PLoS Pathog. 2021 Dec 1;17(12):e1010116. doi: 10.1371/journal.ppat.1010116 (PMC8668125; doi:10.1371/journal.ppat.1010116)
Supplement: S1 Text — Fig A. Sequence alignment of VgrG proteins in A. dhakensis SSU. The three VgrG proteins were aligned using the BLAST Multiple Alignment tool. Alignment was downloaded in Clustal format and visualized using ESPript with default settings (https://espript.ibcp.fr). The predicted structure of VgrG1, generated by Phyre2, was used as structural template in ESPript. Fig B. Pull-down analyses of A. dhakensis effector-structural protein interactions. a, Pull-down analysis of structural proteins with the catalytically inactive TseIHFH-AAA. The full-length protein and the cleaved C-terminus of TseI are indicated. b, Pull-down analysis of structural proteins with the effector TseC. c, Pull-down analysis of TssA with TseIHFH-AAA. d, Pull-down analysis of TssA with TseC. For all pull-down assays, effectors carry a C-terminal 3V5 tag and bait proteins are fused with an N-terminal 6His tag. His-sfGFP serves as a negative control for nonspecific interaction. All proteins were individually expressed in E. coli and cell lysates mixed in pairs. Samples were detected by Western blotting analysis using the anti-V5 and anti-His antibodies, respectively. All pull-down experiments were performed at least twice and a representative result is shown. Fig C. Pull-down analyses of V. cholerae effector-structural protein interactions. a, Pull-down analysis of V. cholerae structural proteins with the catalytically inactive TseLD425A. b, Pull-down analysis of V. cholerae structural proteins with the effector VasX. c, Pull-down analysis of V. cholerae TssA with TseLD425A. d, Pull-down analysis of V. cholerae TssA with VasX. For all pull-down assays, effectors carry a C-terminal 3V5 tag and bait proteins are fused with an N-terminal 6His tag. His-sfGFP serves as a negative control for nonspecific interaction. All proteins were individually expressed in E. coli and cell lysates mixed in pairs. Samples were detected by Western blotting analysis using the anti-V5 and anti-His antibodies, respectively. A [file ppat.1010116.s001.docx]

# Supporting Information

**Fig A. Sequence alignment of VgrG proteins in *A. dhakensis* SSU.** The three VgrG proteins were aligned using the BLAST Multiple Alignment tool. Alignment was downloaded in Clustal format and visualized using ESPript with default settings (<https://espript.ibcp.fr>). The predicted structure of VgrG1, generated by Phyre2, was used as structural template in ESPript.

**Fig B. Pull-down analyses of *A. dhakensis* effector-structural protein interactions. a**, Pull-down analysis of structural proteins with the catalytically inactive TseI^HFH-AAA^. The full-length protein and the cleaved C-terminus of TseI are indicated. **b**, Pull-down analysis of structural proteins with the effector TseC. **c**, Pull-down analysis of TssA with TseI^HFH-AAA^. **d**, Pull-down analysis of TssA with TseC. For all pull-down assays, effectors carry a C-terminal 3V5 tag and bait proteins are fused with an N-terminal 6His tag. His-sfGFP serves as a negative control for nonspecific interaction. All proteins were individually expressed in *E. coli* and cell lysates mixed in pairs. Samples were detected by Western blotting analysis using the anti-V5 and anti-His antibodies, respectively. All pull-down experiments were performed at least twice and a representative result is shown.

**Fig C. Pull-down analyses of *V. cholerae* effector-structural protein interactions. a,** Pull-down analysis of *V. cholerae* structural proteins with the catalytically inactive TseL^D425A^. **b,** Pull-down analysis of *V. cholerae* structural proteins with the effector VasX. **c**, Pull-down analysis of *V. cholerae* TssA with TseL^D425A^. **d**, Pull-down analysis of *V. cholerae* TssA with VasX. For all pull-down assays, effectors carry a C-terminal 3V5 tag and bait proteins are fused with an N-terminal 6His tag. His-sfGFP serves as a negative control for nonspecific interaction. All proteins were individually expressed in *E. coli* and cell lysates mixed in pairs. Samples were detected by Western blotting analysis using the anti-V5 and anti-His antibodies, respectively. All pull-down experiments were performed at least twice and a representative result is shown.

**Table A. Plasmids, strains and primers.**

**
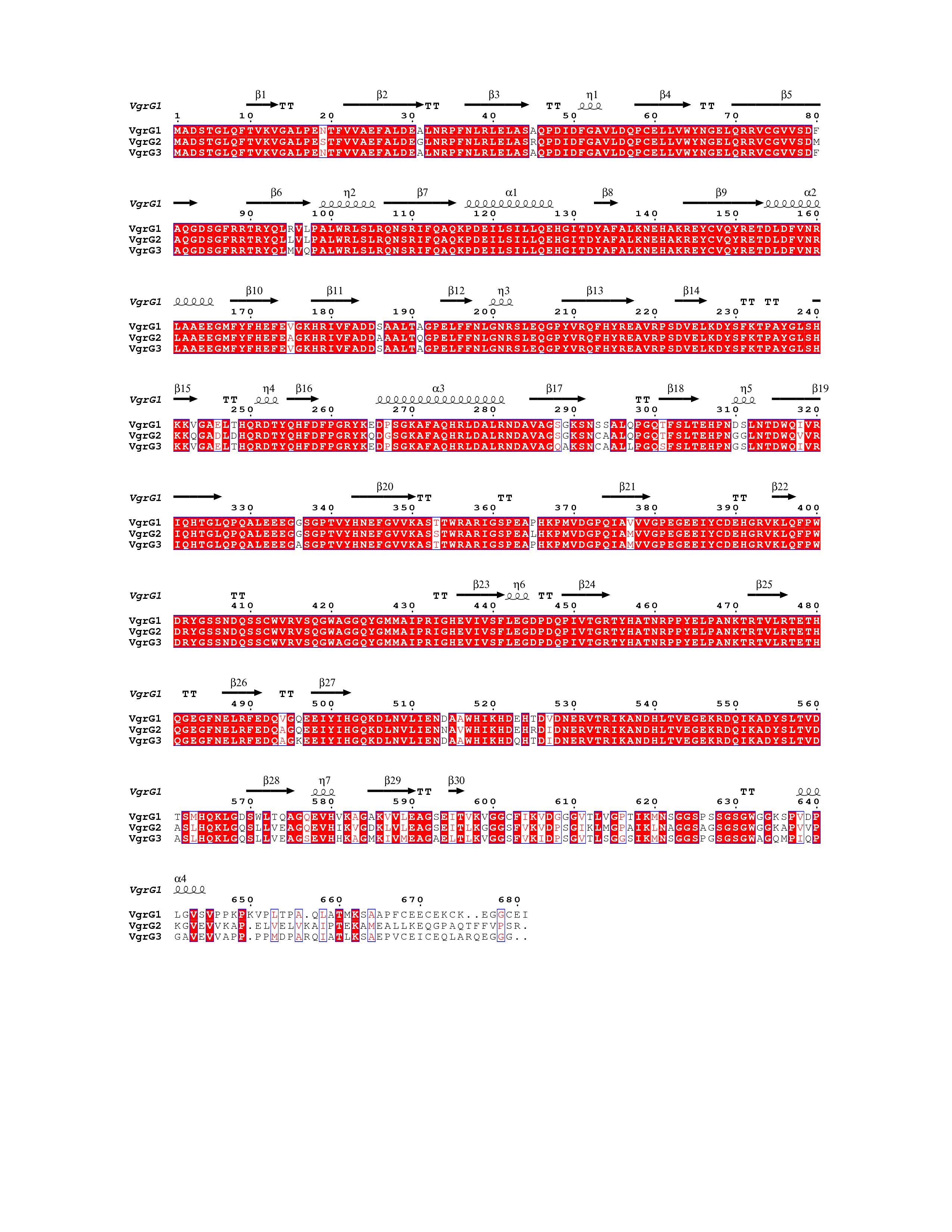
**

**Fig A. Sequence alignment of VgrG proteins in *A. dhakensis* SSU.** The three VgrG proteins were aligned using the BLAST Multiple Alignment tool. Alignment was downloaded in Clustal format and visualized using ESPript with default settings (<https://espript.ibcp.fr>). The predicted structure of VgrG1, generated by Phyre2, was used as structural template in ESPript.

**
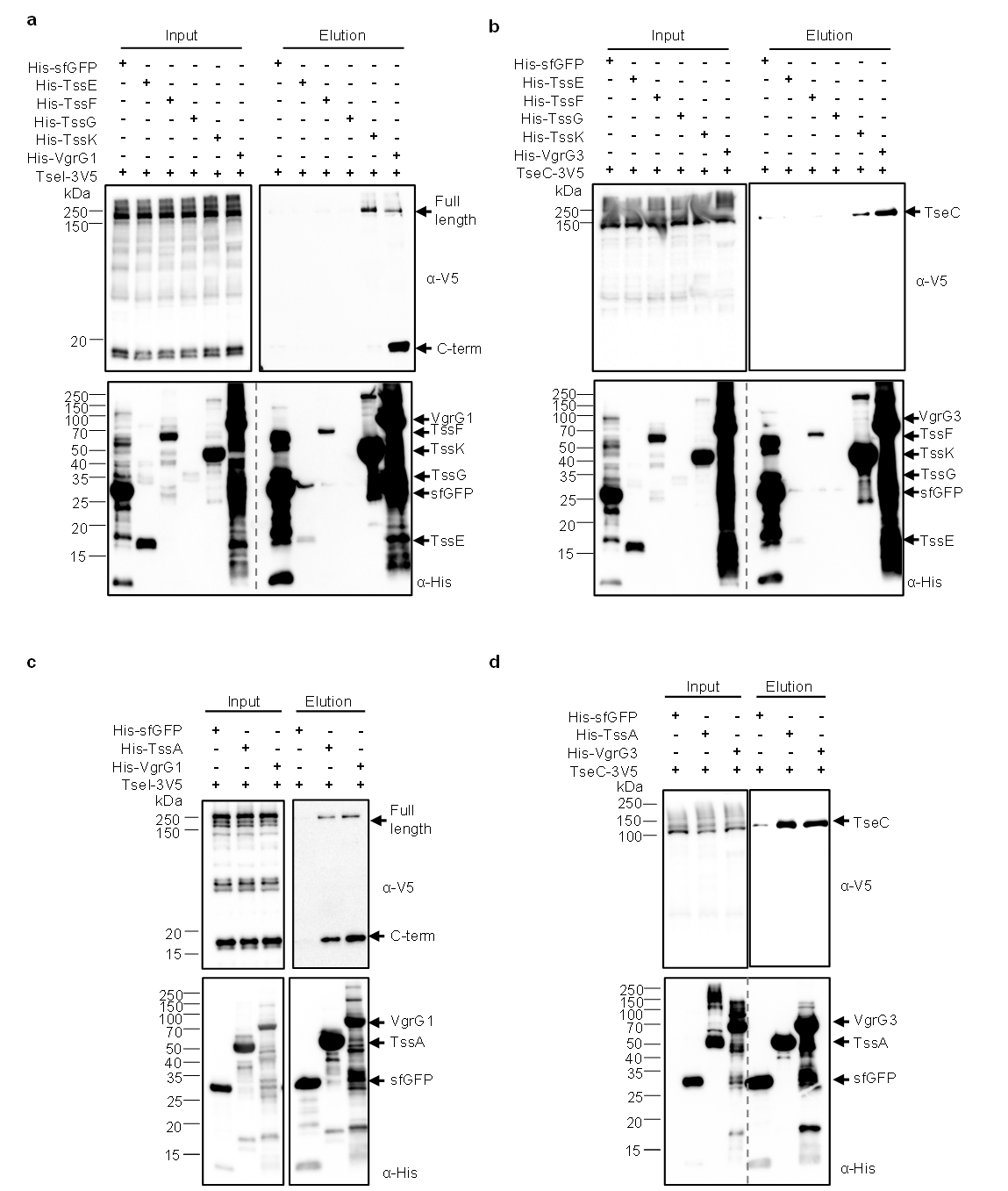
**

**Fig B. Pull-down analyses of *A. dhakensis* effector-structural protein interactions. a**, Pull-down analysis of structural proteins with the catalytically inactive TseI^HFH-AAA^. The full-length protein and the cleaved C-terminus of TseI are indicated. **b**, Pull-down analysis of structural proteins with the effector TseC. **c**, Pull-down analysis of TssA with TseI^HFH-AAA^. **d**, Pull-down analysis of TssA with TseC. For all pull-down assays, effectors carry a C-terminal 3V5 tag and bait proteins are fused with an N-terminal 6His tag. His-sfGFP serves as a negative control for nonspecific interaction. All proteins were individually expressed in *E. coli* and cell lysates mixed in pairs. Samples were detected by Western blotting analysis using the anti-V5 and anti-His antibodies, respectively. All pull-down experiments were performed at least twice and a representative result is shown.

**
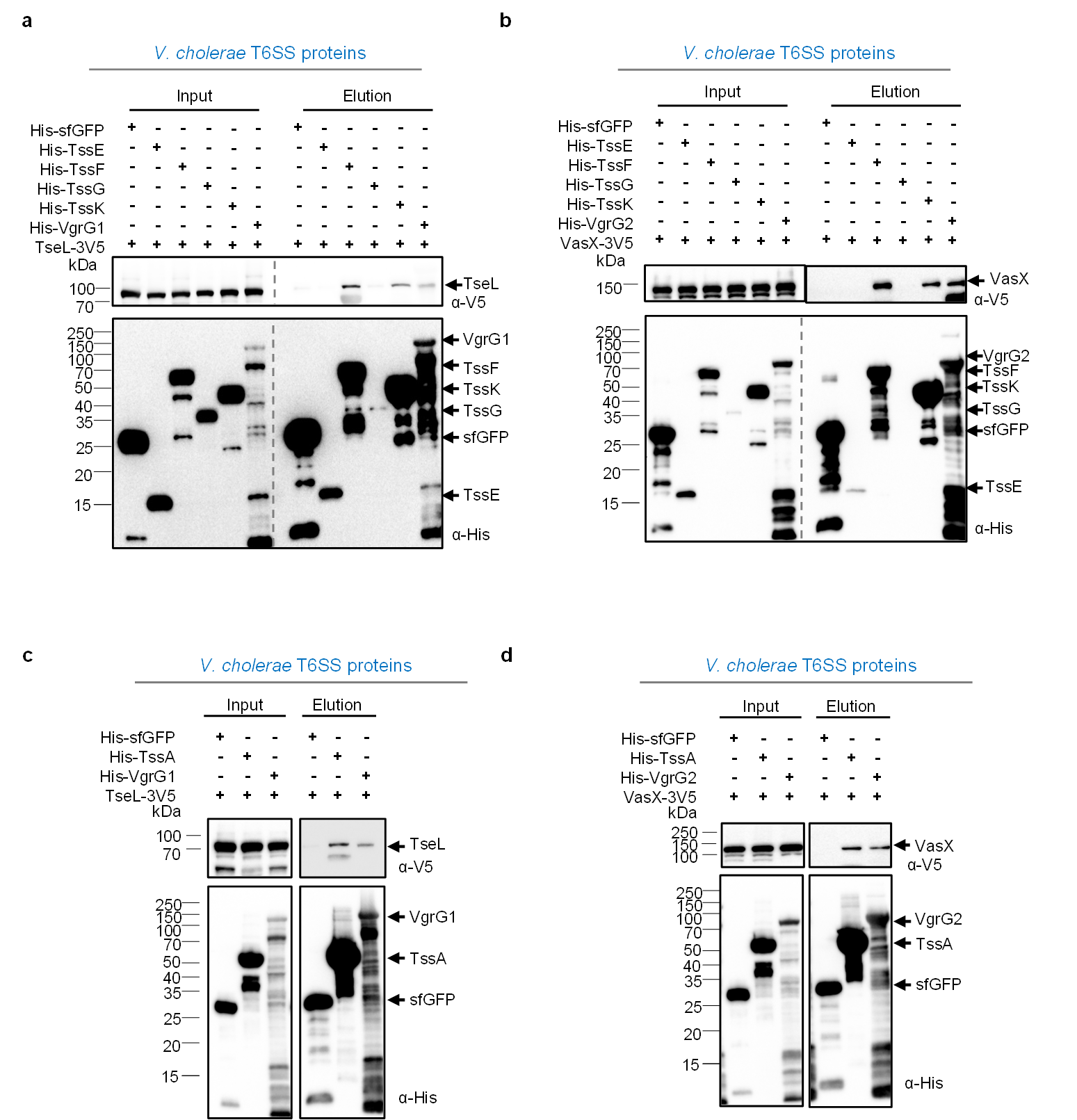
**

**Fig C. Pull-down analyses of *V. cholerae* effector-structural protein interactions. a,** Pull-down analysis of *V. cholerae* structural proteins with the catalytically inactive TseL^D425A^. **b,** Pull-down analysis of *V. cholerae* structural proteins with the effector VasX. **c**, Pull-down analysis of *V. cholerae* TssA with TseL^D425A^. **d**, Pull-down analysis of *V. cholerae* TssA with VasX. For all pull-down assays, effectors carry a C-terminal 3V5 tag and bait proteins are fused with an N-terminal 6His tag. His-sfGFP serves as a negative control for nonspecific interaction. All proteins were individually expressed in *E. coli* and cell lysates mixed in pairs. Samples were detected by Western blotting analysis using the anti-V5 and anti-His antibodies, respectively. All pull-down experiments were performed at least twice and a representative result is shown.

**Table A. Plasmids, strains and primers.**

| **Plasmid** | **Description** | **Reference** |
| --- | --- | --- |
| pDS132 | Suicidal conjugation vector for all chromosomal allelic changes | (1) |
| pDS132-*vgrG1* | Suicidal vector to construct in-frame deletion mutant of SSU *vgrG1* | (2) |
| pDS132-*vgrG2* | Suicidal vector to construct in-frame deletion mutant of SSU *vgrG2* | (3) |
| pDS132-*vgrG3* | Suicidal vector to construct in-frame deletion mutant of SSU *vgrG3* | (4) |
| pDS132-*tecC* | Suicidal vector to construct in-frame deletion mutant of SSU *tecC* | This study |
| pDS132-*tecI* | Suicidal vector to construct in-frame deletion mutant of SSU *tecI* | This study |
| pDS132-*tecL* | Suicidal vector to construct in-frame deletion mutant of V52 *tecL* | This study |
| pDS132-*vasW* | Suicidal vector to construct in-frame deletion mutant of V52 *vasW* | This study |
| pBAD18Kan | Arabinose inducible expression | (5) |
| pBAD18Cm | Arabinose inducible expression | Lab stock |
| pBAD24Kan | Arabinose inducible expression | Lab stock |
| pBAD18Kan-SSU VgrG1 | Arabinose inducible expression of SSU VgrG1 with a C-terminal 6×His tag | This study |
| pBAD18Kan-SSU VgrG2 | Arabinose inducible expression of SSU VgrG2 | This study |
| pBAD18Kan-SSU VgrG3 | Arabinose inducible expression of SSU VgrG3 with a C-terminal 6×His tag | This study |
| pBAD18Kan-SSU VgrG3^1TL^ | Arabinose inducible expression of SSU VgrG3^1TL^ with a C-terminal 6×His tag | This study |
| pBAD18Kan-SSU VgrG3^2TL^ | Arabinose inducible expression of SSU VgrG3^2TL^ | This study |
| pBAD18Kan-SSU VgrG3^VC-2TL^ | Arabinose inducible expression of SSU VgrG3 ^VC-2TL^ | This study |
| pBAD18Cm-TsiV2 | Arabinose inducible expression of TsiV2 | (4) |
| pET28a-sfGFP | IPTG inducible expression of sfGFP with an N-terminal 6×His tag | (2) |
| pETDuet2-His-TssA | IPTG inducible expression of V52 TssA with an N-terminal 6×His tag | (6) |
| pET28a-His-VCVgrG1 | IPTG inducible expression of V52 VgrG1 with an N-terminal 6×His tag | This study |
| pBAD24Kan-TseL^D425A^-3V5 | Arabinose inducible expression of TseL^D425A^ with a C-terminal 3V5 tag | This study |
| pET28a-His-VCVgrG2 | IPTG inducible expression of V52 VgrG2 with an N-terminal 6×His tag | This study |
| pBAD18Cm-VasX-3V5 | Arabinose inducible expression of VasX with a C-terminal 3V5 tag | (7) |
| pETDuet1-His-SSUVgrG1 | IPTG inducible expression of SSU VgrG1 with an N-terminal 6×His tag | (2) |
| pBAD24Kan-TseI^AAA^-3V5 | Arabinose inducible expression of TseI^AAA^ with a C-terminal 3V5 tag | (2) |
| pET28a-His-SSUTssA | IPTG inducible expression of SSU TssA with an N-terminal 6×His tag | This study |
| pETDuet1-His-SSUVgrG3 | IPTG inducible expression of SSU VgrG3 with an N-terminal 6×His tag | This study |
| pBAD18Cm-TseC-3V5 | Arabinose inducible expression of TseC with a C-terminal 3V5 tag | (4) |
| pET28a-His-SSUTssE | IPTG inducible expression of SSU TssE with an N-terminal 6×His tag | This study |
| pET28a-His-SSUTssF | IPTG inducible expression of SSU TssF with an N-terminal 6×His tag | This study |
| pET28a-His-SSUTssG | IPTG inducible expression of SSU TssG with an N-terminal 6×His tag | This study |
| pET28a-His-SSUTssK | IPTG inducible expression of SSU TssK with an N-terminal 6×His tag | This study |
| pET28a-His-VCTssE | IPTG inducible expression of V52 TssE with an N-terminal 6×His tag | This study |
| pET28a-His-VCTssF | IPTG inducible expression of V52 TssF with an N-terminal 6×His tag | This study |
| pET28a-His-VCTssG | IPTG inducible expression of V52 TssG with an N-terminal 6×His tag | This study |
| pET28a-His-VCTssK | IPTG inducible expression of V52 TssK with an N-terminal 6×His tag | This study |
| pBAD24Kan-FLAG-TseP^E663A^ | Arabinose inducible expression of TseP^E663A^ with an N-terminal FLAG tag | This study |
| pBAD24Cm -TseC^∆14^-FLAG | Arabinose inducible expression of TseC^∆14^ with a C-terminal FLAG tag | This study |
| pBAD24Kan -VasX^∆16^-FLAG | Arabinose inducible expression of VasX^∆16^ with a C-terminal FLAG tag | This study |
| pBAD24Kan -TseL^D425A^-FLAG | Arabinose inducible expression of TseL^D425A^ with a C-terminal FLAG tag | This study |

| **Strain** | **Genotype** | **Description** | **Reference** |
| --- | --- | --- | --- |
| *Aeromonas dhakensis* SSU | Parental | Parental strain, streptomycin resistant | (4) |
|  | Δ*vasK* | T6SS null, in-frame deletion of *vasK* | (4) |
|  | Δ*tseP* | In-frame deletion of *tseP* | (3) |
|  | Δ*vgrG1* | In-frame deletion of *vgrG1* | (2) |
|  | Δ*vgrG2* | In-frame deletion of *vgrG2* | (3) |
|  | Δ*vgrG3* | In-frame deletion of *vgrG3* | (4) |
|  | Δ*tseI^ei^* | In-frame deletion of toxin-coding sequence of *tseI* and *tsiI* | (2) |
|  | *ΔtseP^ei^* | In-frame deletion of toxin-coding sequence of *tseP* and *tsiP* | (3) |
|  | *ΔtseC^ei^* | In-frame deletion of toxin-coding sequence of *tseC* and *tsiC* | (4) |
|  | Δ*vgrG1&2* | In-frame deletion of *vgrG1* and *vgrG2* | This study |
|  | Δ*vgrG2&3* | In-frame deletion of *vgrG2* and *vgrG3* | This study |
|  | Δ*vgrG1&3* | In-frame deletion of *vgrG1* and *vgrG3* | This study |
|  | Δ*vgrG1&2&3* | In-frame deletion of all three *vgrG* genes | This study |
|  | Δ*tecI* | In-frame deletion of chaperone gene *tecI* | (2) |
|  | Δ*tecC* | In-frame deletion of chaperone gene *tecC* | (4) |
|  | ∆*tseP tecI* | In-frame deletion of *tseP* and *tecI* | This study |
|  | ∆*tseP tecC* | In-frame deletion of *tseP* and *tecC* | This study |
|  | ∆*tecI tecC* | In-frame deletion of *tecI* and *tecC* | This study |
|  | ∆*tseP tecI tecC* | In-frame deletion of *tseP, tecI* and *tecC* | This study |
| *Vibrio cholerae* V52 | Parental | Deletion in *rtxA hlyA hapA*, parental strain | (8) |
|  | Δ*vasK* | T6SS null, in-frame deletion of VCA0120 | (8) |
|  | Δ*vasW* | In-frame deletion of VCA0019 | This study |
|  | Δ*tecL* | In-frame deletion of VC1417 | (4) |
|  | Δ*vgrG3^ei^* | In-frame deletion of VCA0123-24 | (9) |
|  | Δ*vgrG3^ei^ vasW* | In-frame deletion of VCA0123-24 and *vasW* | This study |
|  | Δ*vgrG3^ei^ tecL* | In-frame deletion of VCA0123-24 and *tecL* | This study |
|  | Δ*vasW tecL* | In-frame deletion of *vasW* and *tecL* | This study |
|  | Δ*vgrG3^ei^ vasW tecL* | In-frame deletion of VCA0123-24, *vasW* and *tecL* | This study |
| *E. coli* |  |  |  |
| T-Fast | F- *proA+B+ lacIq* Δ*lacZM15 / fhuA2* Δ*(lac-proAB) glnV galK16 galE15 R(zgb-210::Tn10)TetS endA1 thi-1* Δ*(hsdS-mcrB)5* | Strain used for cloning and gene expression | TIANGEN |
| DH5alpha | F- *Φ80lacZΔM15 Δ(lacZYA-argF) U169 recA1 endA1 hsdR17 phoA supE44 thi-1 gyrA96 relA1* *λ-* | Strain used for cloning and gene expression | Invitrogen |
| PIR1 | F- *∆lac169 rpoS(Am) robA1 creC510 hsdR514 endA recA1 uidA(∆MluI)::pir-116* | Strain used for cloning | Invitrogen |
| WM6026 | *lacI^q^*, *rrnB3*, Δ*lacZ*4787, *hsdR514*, Δ*araBAD567*, Δ*rhaBAD568*, *rph-1*, *attl*::pAE12(Δ*ori*R6K-*cat*::Frt5), Δ*endA*::Frt, *uidA*(Δ*MluI*)::*pir*, *att*HK::pJK1006Δ(*ori*R6K-*cat*::Frt5; *trfA*::Frt) | Strain used for conjugation, diaminopimelic acid auxotroph | Mekalanos lab |
| BL21(DE3) | F^–^ *ompT* *gal* *dcm* *lon* *hsdS_B_*(*r_B_*^–^*m_B_*^–^) λ(DE3 [*lacI* *lacUV5*-*T7p07* *ind1* *sam7* *nin5*]) [*malB*^+^]_K-12_(λ^S^) | Strain used for protein expression | Lab stock |
| SM10 (λ pir) | Km^R^ , *thi-1, thr, leu, tonA, lacY, supE, recA::RP4-2- Tc::Mu, pir* | Strain used for conjugation | Mekalanos lab |
| CC114 |  | A transposon mutant used as prey for T6SS killing | Mekalanos lab |
| MG1655 | K-12 F- *λ- ilvG- rfb-50 rph-1* | Strain used for competition assay | Lab stock |

| **Primer** | **Sequence (5’-3’)** | **Description** |
| --- | --- | --- |
| 2404-NotI-F | gcggccgcaATGGCAGACAGCACAGGATTAC | Forward primer to amplify SSUVgrG3 |
| 2404-KpnI-R | ggtacctcaGCCACCACCCTCCTGTCT | Reverse primer to amplify SSUVgrG3 |
| NdeI-VC1416-F | ttCATatggcgacattagcgtacagcattg | Forward primer to amplify VCVgrG1 |
| VC1416-EcoRI-R | cgaattcTTATCaagcaataatgcgttgccattcttg | Reverse primer to amplify VCVgrG1 |
| NdeI-VCA0018-F | ttCATatggcgacattagcgtacagcattg | Forward primer to amplify VCVgrG2 |
| VCA0018-EcoRI-R | cgaattcTCAttaatttcccttggcctcttcacacac | Reverse primer to amplify VCVgrG2 |
| NdeI-VCA0109-F | ccatatgacgtacatcgcacctgaagagagtgc | Forward primer to amplify VCTssE |
| VCA0109-HindIII-R | caagcttTTATCAaaacactcgatattttctgctttgatccaataacag | Reverse primer to amplify VCTssE |
| NdeI-VCA0111-F | gccatatggggcacacagagcggaatg | Forward primer to amplify VCTssG |
| VCA0111-HindII-R | caagcttTTATCAttgccttacttgaattaacacatgtttattggc | Reverse primer to amplify VCTssG |
| hifi-NdeI-VCA0114-F | tgccgcgcggcagccatatgtttgcgcgtaaccgagtgatct | Forward primer to amplify VCTssK |
| VCA0114-EcoRI-R | cgaattcTTATCAgctcctgatagcccagaattgcag | Reverse primer to amplify VCTssK |
| hifi-NdeI-VCA0110-F | tgccgcgcggcagccatatgacgcaagacaagtatttcagggaagagc | Forward primer to amplify VCTssF |
| VCA0110-EcoRI-R | cgaattcTTAtcagatcaacggctgcattccg | Reverse primer to amplify VCTssF |
| NheI-SSU932-F | tggctagcccgcatctctcttcctgggatag | Forward primer to amplify SSUTssE |
| SSU932-XhoI-R | tgctcgagTTAtcagaccatgcggtagtgacgg | Reverse primer to amplify SSUTssE |
| NheI-SSU933-F | tgGCTAGCtcgctggaacattacttcagggatg | Forward primer to amplify SSUTssF |
| SSU933-XhoI-R | tgctcgagTTAtcacatcagggagtgctgaccg | Reverse primer to amplify SSUTssF |
| NheI-SSU934-F | tgGCTAGCgtggccagtccagatcggtc | Forward primer to amplify SSUTssG |
| SSU934-XhoI-R | tgctcgagTTATCAttcctgcacacaaatggtcacg | Reverse primer to amplify SSUTssG |
| NheI-SSU937-F | tgGCTAGCATGtcgagtcgaaatcgggttatctg | Forward primer to amplify SSUTssK |
| SSU937-XhoI-R | tgctcgagTTAtcactggctgcgaatggccc | Reverse primer to amplify SSUTssK |
| NheI-SSU942-F | tgGCTAGCatgagctatcaacacccctggtgtg | Forward primer to amplify SSUTssA |
| SSU942-XhoI-R | tgctcgagTTAtcatttcgacaacggcgcc | Reverse primer to amplify SSUTssA |
| pDS132-SSU-d927-KO1 | tgatgggttaaaaaggatcgaacaagacccgtactgtgctgc | Forward primer to amplify the upstream of SSU *tecI* for constructing ∆*tecI* |
| pDS132-SSU-d927-KO2 | ctggaaacagaggggctgattgcctctgtatc | Reverse primer to amplify the upstream of SSU *tecI* for constructing ∆*tecI* |
| pDS132-SSU-d927-KO3 | caatcagcccctctgtttccagcgctggaagc | Forward primer to amplify the downstream of SSU *tecI* for constructing ∆*tecI* |
| pDS132-SSU-d927-KO4 | gcatgcggtacctctagaagcactgttcttggccatggagg | Reverse primer to amplify the downstream of SSU *tecI* for constructing ∆*tecI* |
| pDS132-SSU-d927-KO5 | gcccgccctacgagttgc | Forward confirmation primer of SSU ∆*tecI* |
| pDS132-SSU-d927-KO6 | aggtgaccagtcgcatgacc | Reverse confirmation primer of SSU ∆*tecI* |
| pDS132-SSU-d2403-KO1 | gtgatgggttaaaaaggatcgcgaggtagcgatccccaaga | Forward primer to amplify the upstream of SSU *tecC* for constructing ∆*tecC* |
| pDS132-SSU-d2403-KO2 | cgtcccgacatcgaactgaacgatcaagacgcatgagtacgcc | Reverse primer to amplify the upstream of SSU *tecC* for constructing *∆tecC* |
| pDS132-SSU-d2403-KO3 | gttcagttcgatgtcgggacg | Forward primer to amplify the downstream of SSU *tecC* for constructing *∆tecC* |
| pDS132-SSU-d2403-KO4 | gcatgcggtacctctagaagccacgaggtcattgtctcgtttc | Reverse primer to amplify the downstream of SSU *tecC* for constructing *∆tecC* |
| pDS132-SSU-d2403-KO5 | ggagttgatccgcaccatcc | Forward confirmation primer of SSU *∆tecC* |
| pDS132-SSU-d2403-KO6 | tatggcagctccaacgacca | Reverse confirmation primer of SSU *∆tecC* |
| pDS132-V52-dvc1417-KO1 | gtgatgggttaaaaaggatcgacagtgggtgttgctgctaa | Forward primer to amplify the upstream of V52 *tecL* for constructing ∆*tecL* |
| pDS132-V52-dvc1417-KO2 | cagagcgagagcaagccaatagacgtttgatgtct | Reverse primer to amplify the upstream of V52 *tecL* for constructing *∆tecL* |
| pDS132-V52-dvc1417-KO3 | ctattggcttgctctcgctctgcatgctttaactcg | Forward primer to amplify the downstream for constructing *∆tecL* |
| pDS132-V52-dvc1417-KO4 | gcatgcggtacctctagaagaatcacagccgccatacctg | Reverse primer to amplify the downstream of V52 *tecL* for constructing *∆tecL* |
| pDS132-V52-dvc1417-KO5 | agtgaagacggacgctttgt | Forward confirmation primer of V52 ∆*tecL* |
| pDS132-V52-dvc1417-KO6 | gatttgcgcagcatcaaggt | Reverse confirmation primer of V52 *∆tecL* |
| pDS132-V52-dvca0019-KO1 | tgatgggttaaaaaggatcgtacgctcgtcaaactcgactgc | Forward primer to amplify the upstream of V52 *vasW* for constructing ∆*vasW* |
| pDS132-V52-dvca0019-KO2 | ctctgcggctggaaccaagctcacccagtt | Reverse primer to amplify the upstream of V52 *vasW* for constructing ∆*vasW* |
| pDS132-V52-dvca0019-KO3 | cttggttccagccgcagagctggcttatca | Forward primer to amplify the downstream of V52 *vasW* for constructing ∆*vasW* |
| pDS132-V52-dvca0019-KO4 | gcatgcggtacctctagaagggccaattttgccgtagcac | Reverse primer to amplify the downstream of V52 *vasW* for constructing ∆*vasW* |
| pDS132-V52-dvca0019-KO5 | aaccaacacctgacggtcga | Forward confirmation primer of V52 ∆*vasW* |
| pDS132-V52-dvca0019-KO6 | atgcccttcactggctaaatgatg | Reverse confirmation primer of V52 ∆*vasW* |
| SSU946-rbs-kpnI-f | CATGGTACCAGGAGGAAACGatggcagacagcacaggattaca | Forward primer to amplify the upstream of SSU *vgrG2* |
| SSU946-XbaI-r | GCTTCTAGATCaTcTggatggcacgaaaaaggtctg | Reverse primer to amplify the upstream of SSU *vgrG2* |
| SSUVgrG3-p1 | tagcGGTACCaggaggaaacgATGGCAGACAGCACAGGATTAC | Forward primer to amplify VgrG3, for joining with the tail sequence by Gibson assembly |
| SSUVgrG3-p2 | CGGTGGCGCCACCACTTC | Reverse primer to amplify VgrG3, for joining with the tail sequence by Gibson assembly |
| SSUVgrG3^1TL^-fd | GGTTGAAGTGGTGGCGCCACCGaaggtgccgctcactcct | Forward primer to amplify VgrG1 tail, for joining with VgrG3 by Gibson assembly |
| SSUVgrG3^1TL^-rv | TGGTGATGGCTGCTTCTAGAaatttcacagccaccctcct | Reverse primer to amplify VgrG1 tail, for joining with VgrG3 by Gibson assembly |
| SSUVgrG3^2TL^-fd | GTTGAAGTGGTGGCGCCACCGgaactggtcgagctggtca | Forward primer to amplify VgrG2 tail, for joining with VgrG3 by Gibson assembly |
| SSUVgrG3^2TL^-rv | TGATGGTGATGGCTGCTTCTAGAgcgggatggcacgaaaa | Reverse primer to amplify VgrG2 tail, for joining with VgrG3 by Gibson assembly |
| VgrG3^VC-2TL^-rv | ggcattgccccggcccaaccggagc | Reverse primer to amplify VgrG3, for joining with the VC-tail sequence by Gibson assembly |
| VgrG3^VC-2TL^-fd | ggttgggccgggaaaatggccgagttgcca | Forward primer to amplify VC-VgrG2tail-VasX-TsiV2 sequence, for joining with SSU VgrG3 by Gibson assembly |
| VgrG3^VC-2TL^-rv2 | GGTGATGGCTGCTTCTAGActattcctcttttaattcttg | Reverse primer to amplify VC-VgrG2tail-VasX-TsiV2 sequence, for joining with SSU VgrG3 by Gibson assembly |
| vasX-pbad24cm-hifi-f | caggaggaaacgatgtctagaagtaatcccaatcaagctgcgaaaac | Forward primer to amplify VC VasX |
| vasX-flag-hifi-r | TAATCTgccgcggtaccagaaccttttcctacaacgagatttcttgtg | Reverse primer to amplify VC VasX |
| 2402-pbad24T-hifi-f | caggaggaaacgatgtctagaagtacgcccaatcaagccg | Forward primer to amplify SSU TseC |
| 2402-flag-rv | CTTGTCATCGTCGTCCTTGTAATCTgccgcggtaccagattcgtg | Reverse primer to amplify SSU TseC |
| 1418 hifi-F | ttgggctagcaggaggtaccatggattcatttaattattgcgtgcagtg | Forward primer to amplify VC TseL |
| 1418-flag-rv | CTTGTCATCGTCGTCCTTGTAATCaccGCATGCtcttatttgcaccttg | Reverse primer to amplify VC TseL |
| 947-pbad24-hifi-f | gGATTACAAGGACGACGATGACAAGatgctcaatggtgagccAggTg | Forward primer to amplify SSU TseP |
| 947-pbad24-hifi-r | AAGGAGAGGATTAGGAATAGGTTTACctcactctctcgccccatcaatac | Reverse primer to amplify SSU TseP |

**References for strains and plasmids.**

1. Philippe N, Alcaraz J-P, Coursange E, Geiselmann J, Schneider D. 2004. Improvement of pCVD442, a suicide plasmid for gene allele exchange in bacteria. Plasmid 51:246–255.

2. Pei T-T, Li H, Liang X, Wang Z-H, Liu G, Wu L-L, et al. 2020. Intramolecular chaperone-mediated secretion of an Rhs effector toxin by a type VI secretion system. Nat Commun 11:1865.

3. Liang X, Pei T-T, Wang Z-H, Xiong W, Wu L-L, Xu P, et al. 2021. Characterization of lysozyme-like effector TseP reveals the dependence of type VI secretion system (T6SS) secretion on effectors in *Aeromonas dhakensis* strain SSU. Appl Environ Microbiol 87:e0043521.

4. Liang X, Moore R, Wilton M, Wong MJQ, Lam L, Dong TG. 2015. Identification of divergent type VI secretion effectors using a conserved chaperone domain. Proc Natl Acad Sci 112:9106–9111.

5. Guzman LM, Belin D, Carson MJ, Beckwith J. 1995. Tight regulation, modulation, and high-level expression by vectors containing the arabinose PBAD promoter. J Bacteriol 177:4121–4130.

6. Stietz MS, Liang X, Li H, Zhang X, Dong TG. 2020. TssA–TssM–TagA interaction modulates type VI secretion system sheath-tube assembly in *Vibrio cholerae*. Nat Commun 11:5065.

7. Liang X, Kamal F, Pei T-T, Xu P, Mekalanos JJ, Dong TG. 2019. An onboard checking mechanism ensures effector delivery of the type VI secretion system in *Vibrio cholerae*. Proc Natl Acad Sci 116:23292–23298.

8. Ma AT, McAuley S, Pukatzki S, Mekalanos JJ. 2009. Translocation of a *Vibrio cholerae* type VI secretion effector requires bacterial endocytosis by host Cells. Cell Host Microbe 5:234–243.

9. Dong TG, Ho BT, Yoder-Himes DR, Mekalanos JJ. 2013. Identification of T6SS-dependent effector and immunity proteins by Tn-seq in *Vibrio cholerae*. Proc Natl Acad Sci 110:2623–2628.
